# Risk of anaphylaxis on commercial flights

**DOI:** 10.1097/ACI.0000000000001090

**Published:** 2025-07-11

**Authors:** Paul J. Turner

**Affiliations:** National Heart & Lung Institute, Imperial College London, London, UK

**Keywords:** allergic reaction, anaphylaxis, epinephrine, food, in-flight medical event

## Abstract

**Purpose of review:**

Air travel has now returned to prepandemic levels, with over 10.5 billion passengers in 2024. Many of these passengers have food allergies, and there is a perception that allergic reactions are common during commercial flights.

**Recent findings:**

A recent systematic review and meta-analysis reported an incidence of in-flight medical events due to allergic reactions of 0.7 (95% CI 0.4–1.1) events per million passengers. For those with food allergies, the incidence of allergic reactions is around 10–100 times lower than that reported for reactions ‘on the ground’ – equivalent to one reaction per 3600 food-allergic passengers in any 1-year period. Reassuringly, there is no evidence that this rate had increased over the past 30 years, despite significant increases in both the prevalence of food allergy and passenger numbers.

**Summary:**

Allergic reactions during commercial flights are uncommon; however, this is very likely to be confounded by the many precautions food-allergic passengers and their families take when flying. Nonetheless, the data confirm that flying can be safe for those with food allergies. While air travel continues to present numerous challenges to those with food allergy, this can be mitigated by consistent and helpful airline policies, which address the concerns of food-allergic individuals.

## INTRODUCTION

Global passenger demand for commercial air travel has returned to prepandemic levels, with over 10.5 billion passengers in 2024 [1]. The prepandemic increase in passenger numbers (around 7% per year since 2006) [2] has been associated with an increase in the number of in-flight medical events (IMEs) reported by airlines and ground-based medical services (GBMS) [3]. However, few studies have evaluated rates of allergic reactions during commercial flights. This is an important knowledge gap, as there is a perception that air travel is dangerous for food-allergic individuals. Certainly, air travel can be difficult for people with food allergies and their families. Not only do they have to contend with the stress of the airport, but they also worry about having a reaction during the flight, and the limited options available were this to occur [4]. In a recent global survey of 4704 food-allergic passengers and their caregivers, 98% of respondents reported increased anxiety when flying; high anxiety levels were reported by two-thirds of respondents [5^▪▪^]. There is a perception that the risk of allergic reactions is increased when flying [4,5^▪▪^]. The purpose of this review is to evaluate the evidence underlying this perception. 

**Box 1:**
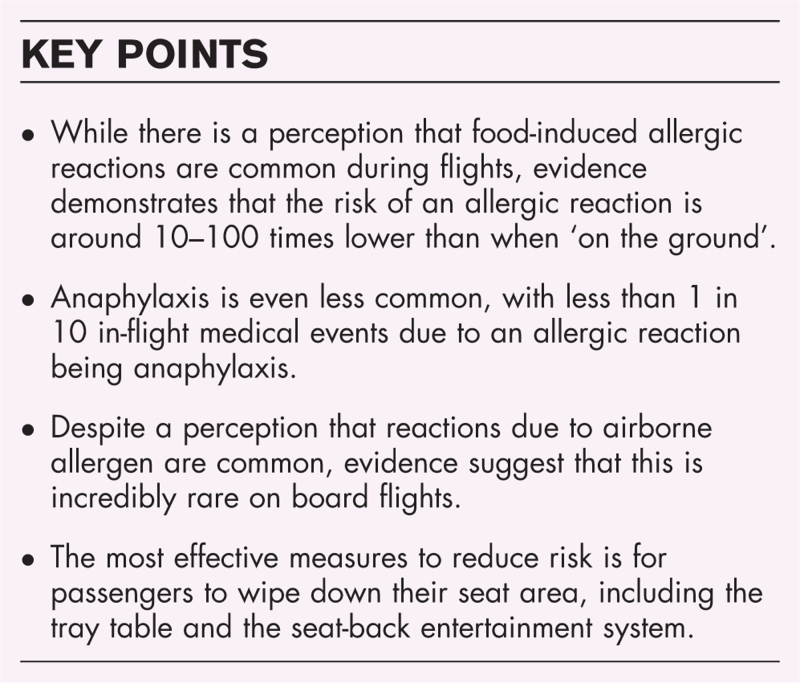
no caption available

## HOW COMMON ARE ALLERGIC REACTIONS ON COMMERCIAL FLIGHTS?

Data to inform the incidence of allergic reactions during commercial flights can be collected from three primary sources:

(1) Airline records: given the high priority of passenger safety during flights, airlines have robust reporting protocols to collect information as to any incident occurring during flights. However, these data are not always made available to the public, nor are all ‘incidents’ reported to cabin crew [5^▪▪^]. Concerns have also been raised in the USA that cabin crew may be selective over what incidents are formally logged [5^▪▪^].
(2) Significant medical incidents are usually reported to GBMS, for insurance reasons. When a medical incident occurs, cabin crew contact a GBMS for advice and support. However, these systems will only capture more significant reactions reported to cabin crew.
(3) A number of surveys have been published in which food-allergic individuals (or their carers) have been asked self-report if they have experienced an allergic reaction during air travel. Such studies tend to be retrospective and subject to a variety of reporting biases, with no adjudication of cases.

## INCIDENCE OF IN-FLIGHT MEDICAL EVENTS DUE TO ALLERGIC REACTIONS

Until recently, there was only one relevant systematic review and meta-analysis (Borges do Nascimento *et al.* in 2021); the authors evaluated the incidence of in-flight medical emergencies due to any cause in studies published between 1945 to November 2020, covering approximately 1.5 billion flights. A sub-analysis identified eight studies, which reported allergic IMEs, with an estimated incidence of 0.64 [95% confidence interval (CI) 0–1.74] IMEs per million passengers [6]. Given these limitations of this data, the UK Civil Aviation Authority (CAA) commissioned a systematic review and meta-analysis, which included studies published from 1980 to December 2022 [7^▪▪^]. Seventeen studies met the inclusion criteria, all of which were assessed as being at low-to-moderate risk of bias. At meta-analysis, the estimated incidence of allergic IMEs was 0.7 (95% CI 0.4–1.1) events per million passengers.

**FIGURE 1:**
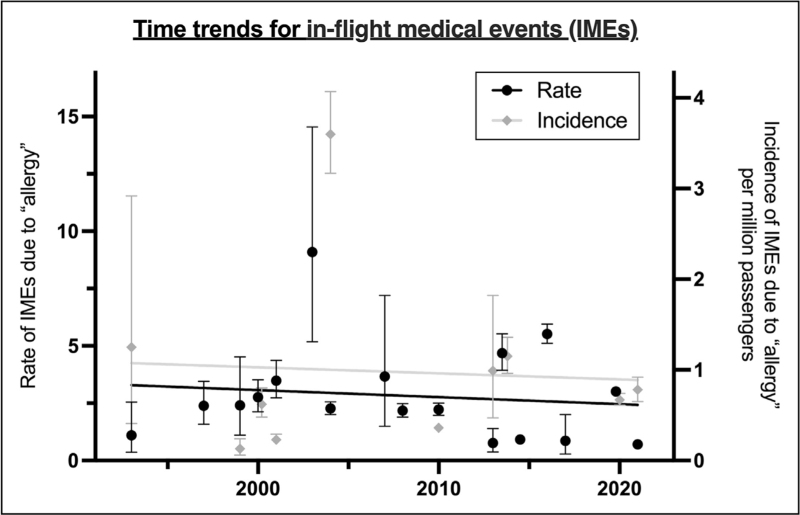
Time trends for in-flight medical events due to allergic causes over the 30 years. Reproduced from reference [7^▪▪^] with permission.

Due to the number of studies included, the authors were also able to assess whether IMEs due to allergic reactions had changed over time. They found no evidence that either the absolute number or proportion of IMEs due to allergic reactions had increased over the past two decades, despite a documented increase in passenger numbers (Fig. 1) [7^▪▪^].

**FIGURE 2:**
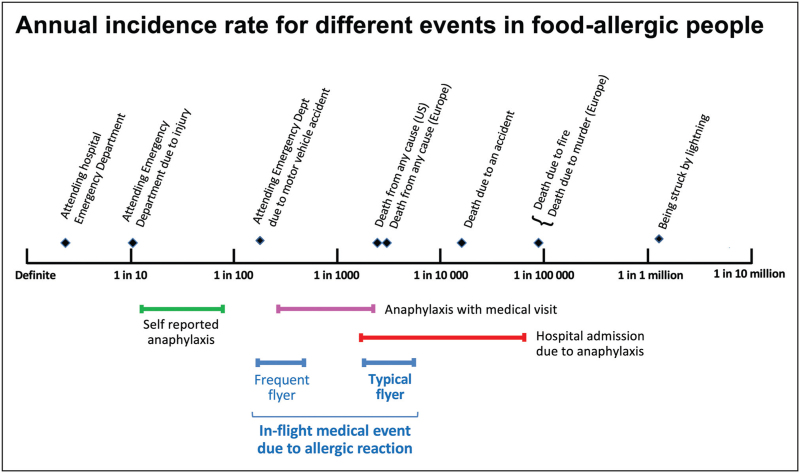
Estimated rates of food-induced allergic reactions in people with known food allergy during commercial flights (assuming a prevalence of 2% for food allergy) compared with equivalent rates when not flying and other risks. Data are shown as 95% confidence intervals. Reproduced from reference [7^▪▪^] with permission.

However, as mentioned above, not all IMEs due to allergic reaction are reported, and it is also useful to convert the above incidence rates to refer to allergic IMEs in passengers at risk of allergic reactions, rather than all passengers. The authors, therefore, went on to estimate the annual incidence of IMEs due to food allergy, assuming a prevalence for food allergy of 2%, that only 50% of food-related IMEs are reported, and that food-allergic passengers fly at the same frequency as those without food allergies. On this basis, the annual incidence of a food-induced allergic reaction was estimated to be 2.7 (95% CI 1.6–4.8) per 10 000 person-years, equivalent to one reaction per 3600 food-allergic passengers travelling in any 1-year period [7^▪▪^]. Compared to the incidence of an unintended allergic reaction due to food ‘on the ground’, the rate of allergic reactions on commercial flights is around 100 times lower than when not flying, and 10 times less frequent than that for medically coded anaphylaxis (Fig. 2).

## FREQUENCY OF UNINTENDED ALLERGIC REACTIONS DURING AIR TRAVEL REPORTED BY AT-RISK INDIVIDUALS

The authors of the CAA report also identified eight studies, which evaluated the frequency of self-reported allergic reactions to peanut/tree nuts in food-allergic individuals while flying [5^▪▪^,^8^–^14^]. These studies were all assessed as being at moderate-high risk of bias, due to the methodology typically used (self-selected respondents, retrospective self-report with no adjudication of cases) [15^▪^]; the exception was a study by Crealey and Byrne [14], which used prospective data collection in an unselected clinical cohort. In general, less than 1 in 10 food-allergic individuals reported at least one incident while flying. Peanut was consistently the most common reported trigger for accidental reactions (Table 1). A high proportion (typically at least half) perceived their reaction as being due to exposure via a noningested route. Respiratory symptoms occurred in between 30 and 50% of reactions, but surveys did not distinguish between upper respiratory symptoms (similar to hay fever) and lower respiratory symptoms (anaphylaxis); where both ‘respiratory symptoms’ and anaphylaxis were reported, the clear discordance between the two indicated a clear predominance of upper respiratory symptoms. In general, only around one-half of incidents were reported to the crew or ground staff.

**Table 1:** Studies describing self-reported allergic reactions to peanut and/or tree nuts during commercial flights

						Reported symptoms					Treatment			Communication	
								
Study	Sample size	Numbers reporting an in-flight allergic reaction	Age of cohort	Due to peanut	Due to noningestion	Anaphylaxis	Respiratory	Cardiovascular	Skin	Gastrointestinal	Adrenaline	Antihistamine	Had own medication	Notified crew	Prenotified airline
Sicherer *et al*.,1999 [8]	3704	42 (1%)	6 months to 50 years	35 (83%)	21 (50%)	5 (12%)	11 (31%)	0	15 (43%)	2 (6%)	6 (14%)	28 (67%)	27 (64%)	14 (33%)	17 (40%)
Comstock *et al*., 2007 [9]	471	45 (10%)	2–50 years	30 (71%)	30 (66%)	36 (88%)	N/A	N/A	N/A	N/A	4 (10%)	15 (37%)	12 (38%)	12 (29%)	N/A
Greenhawt *et al*., 2009 [10]	(150)	150	6 months to 60y	96 (64%)	125 (83%)	50 (33%)	42 (28%)	2 (1.4%)	84 (56%)	11 (7.5%)	15 (10%)	115 (77%)	115 (76%)	67 (44%)	96 (63%)
Greenhawt *et al*., 2013 [11]	3273	346 (11%)	3 months to 50 years	239 (70%)	269 (78%)	N/A	287 (83%)	77 (22%)	290 (84%)	84 (24%)	46 (13%)	297 (86%)	309 (89%)	177 (51%)	193 (56%)
Beaumont *et al*., 2015 [12]	196	12 (6%)	4–47 years	N/A	N/A	5 (42%)	5 (42%)	0	10 (83%)	2 (17%)	6 (50%)	7 (58%)	N/A	9 (75%)	6 (50%)
Martinez-Flores *et al*., 2022 [13]	13200	16 (0.1%)	4–80 years	10 (63%)	7 (44%)	N/A	16 (100%)	6 (38%)	14 (88%)	13 (81%)	3 (19%)	10 (63%)	N/A	N/A	N/A
Crealey *et al*., 2022 [14]	498	3 (0.6%)	Children median 7 years	1 (33%)	0	1 (33%)	1 (33%)	N/A	N/A	N/A	1 (33%)	2 (67%)	N/A	0	N/A
Warren *et al*., 2023 [5^▪▪^]	4704	400 (8.5%)	All ages	46%	N/A	N/A	205 (51%)	67 (17%)	349 (87%)	119 (30%)	60 (15%)	243 (61%)	376 (94%)	238 (60%)	332 (83%)

## HOW COMMON IS ANAPHYLAXIS ON COMMERCIAL FLIGHTS?

Unfortunately, data from airlines and GBMS do not distinguish between anaphylaxis and nonanaphylaxis events. While the first-line treatment of anaphylaxis is an intramuscular injection of adrenaline (epinephrine), it is well established that adrenaline is underused for anaphylaxis, even in the healthcare settings [16]. At the same time, food-allergic individuals (or their carers) may use intramuscular adrenaline to treat more mild, nonanaphylaxis reactions – particularly if access to Emergency Medical Services is challenging. Evidence for this can be seen in Table 1, where there is discordance in the proportion of reactions, which were classified as anaphylaxis and the number treated with intramuscular adrenaline.

To address this evidence gap, Kodoth *et al.* [17^▪^] undertook a retrospective study of the MedAire GBMS database over a 3-year period (2017–2019). A total of 4230 allergic events were identified, of which 398 (9.4%) had adrenaline administration recommended by the GBMS; at least one dose of adrenaline was administered in 328 cases. Using data only from those airlines, which consistently used GBMS to log IMEs, the incidence of allergic events was 0.91 cases per million passengers (not dissimilar to the above estimates, and the incidence of allergic IMEs for which adrenaline administration was recommended was 0.08 (interquartile range 0.02–0.16) cases per million passengers. A limitation of the analysis was the use of adrenaline as a surrogate for anaphylaxis and/or severe allergic reaction; however, it is likely that not every reaction where adrenaline administration was recommended was anaphylaxis, as GBMS are probably cautious in their assessment of potential anaphylaxis, with a lower threshold for recommending adrenaline treatment given the lack of ground-based emergency medical services. The authors concluded that IMEs requiring adrenaline treatment are rare, with a rate of one event in 12.5 million passengers – which is probably an overestimate for the above reasons.

## COMMENTARY

There are two ‘myths’ revealed by the data presented above. Despite the perception that allergic reactions are more common when flying, with up to one in 10 people reporting an in-flight allergic reaction in surveys, more objective evidence suggests that the opposite is true: that the risk of allergic reactions is 10–100 times lower on board commercial flights than when ‘on the ground’. These figures do need to be interpreted in the context of the many precautions food-allergic passengers take when travelling. These range from bringing their own food to eat during the flight to avoiding flying in the first place [4,5▪▪]. Healthcare professionals and airlines need to acknowledge these concerns and address them in a consistent and evidence-based way. In particular, airline policies with respect to food allergies are not always readily available [18,19], and can differ significantly between different carriers. Policies may be implemented inconsistently by cabin crew and ground staff [4,5^▪▪^,^11^], which does not provide reassurance to those with food allergies that their concerns are being taken seriously.

**FIGURE 3:**
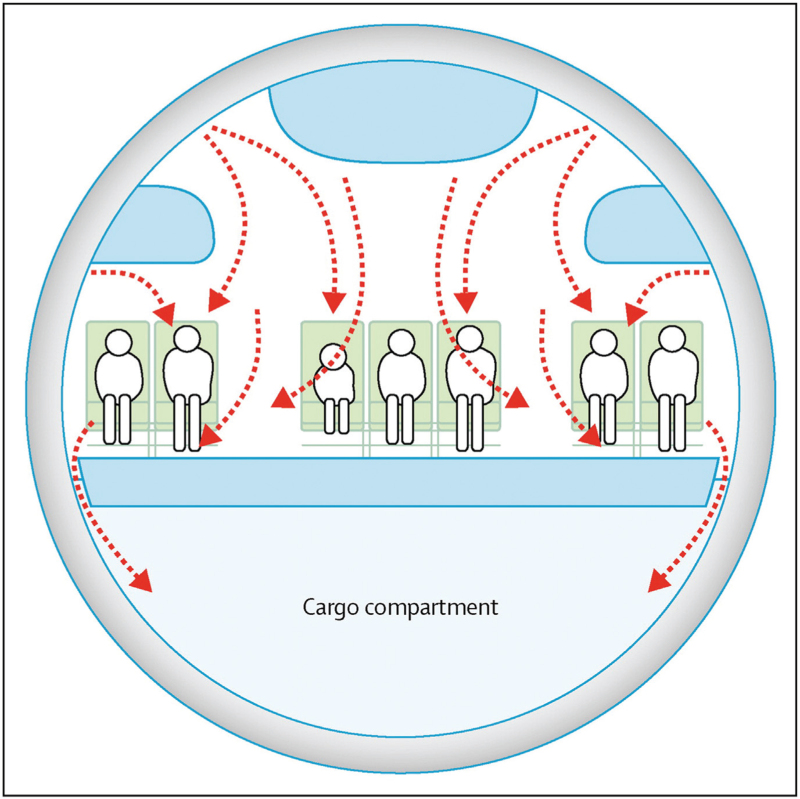
Model of air circulation in a passenger cabin on commercial aircraft. Copyright 2005 Elsevier Ltd. Re-use granted by Elsevier as part of the Elsevier COVID-19 resource centre.

The second ‘myth’ is that reactions due to ‘airborne’ allergens are common. Indeed, the studies shown in Table 1 suggest that over half of reactions are due to noningestion. However, research studies (including aircraft simulations) demonstrate no evidence to support airborne transmission of nut allergens as a likely phenomenon [15^▪^^,^^20▪^]. Modern aircraft have ‘Environmental Control Systems’ (ECS), which provide a safe and comfortable environment for passengers and crew. Air circulates from the top of the cabin, down and across into vents in the floor (Fig. 3). This results in air circulating across the aircraft, rather than along the cabin, minimizing the potential for spreading passenger-generated contaminants through the cabin. In addition, the cabin air is completely exchanged every 3–4 min (in hospital wards, this only happens every 10 min) [21], passing through HEPA filters, which would remove at least 99.97% of any food particles that might be present [20^▪^]. Thus, the ECS does not allow for airborne transmission of food allergens.

Rather, studies have demonstrated that peanut allergy is easily transmitted, both through touch and in saliva [22]. For example, peanut residue can be found on aircraft surfaces (seats, seat-back entertainment systems, trays, and floor) [23,24], due to consumption of peanut on prior flights. This residue can be transmitted to the hands and then transferred either to food being consumed or directly to the individuals mouth/face. This explains the discrepancy between the perception that ‘airborne peanut’ is a common cause of allergic reactions, and study data demonstrating an extremely low risk of reaction due to aerosolized peanut in challenge studies [20^▪^]. Therefore, the most effective measure is for passengers to wipe down their seat area (including tray table and seat-back entertainment system) [[15^▪^,20^▪^]; this can be facilitated through airlines allowing food-allergic passengers to preboard clean their seating area – something already required in the USA by the United States of America Department of Transportation, when requested by a passenger [25].

## CONCLUSION

For a typical food-allergic passenger on a commercial flight, the risk of an allergic reaction is around 10–100 times lower than when ‘on the ground’. However, this needs to be interpreted in the context of food-allergic passengers reporting high levels of anxiety when travelling by air, resulting in them taking significant precautions that are likely to reduce the risk of in-flight allergic reactions. One of the most effective measures to reduce risk is for passengers to wipe down their seat area, including the tray table and the seat-back entertainment system. The proteins, which cause food allergy are often ‘sticky’ and can adhere to these surfaces, from where they are easily transferred to a person's hands and on to food that may be consumed. Airline companies should support passengers in wiping down their seating area, for example, through preboarding.

## Acknowledgements

*None*.

### Financial support and sponsorship

*Funding: this research was supported by the UK Medical Research Council (reference MR/W018616/1) and the UK Civil Aviation Authority.*

### Conflicts of interest

*P.J.T. reports grants from UK Medical Research Council and contracted funding from the UK Civil Aviation Authority for the submitted work; grants from the NIHR/Imperial Biomedical Research Centre and UK Food Standards Agency, outside the submitted work; personal fees from UK Food Standards Agency, outside the submitted work.*

## REFERENCES AND RECOMMENDED READING

Papers of particular interest, published within the annual period of review, have been highlighted as:

▪ of special interest
▪▪ of outstanding interest
